# Dual Plasmon Resonances and Tunable Electric Field in Structure-Adjustable Au Nanoflowers for Improved SERS and Photocatalysis

**DOI:** 10.3390/nano11092176

**Published:** 2021-08-25

**Authors:** Yi-Xin Zhao, Hao-Sen Kang, Wen-Qin Zhao, You-Long Chen, Liang Ma, Si-Jing Ding, Xiang-Bai Chen, Qu-Quan Wang

**Affiliations:** 1 Hubei Key Laboratory of Optical Information and Pattern Recognition, Wuhan Institute of Technology, Wuhan 430205, China; yujin65536@126.com (Y.-X.Z.); kanghaosen2021@126.com (H.-S.K.); zwq1913256258@126.com (W.-Q.Z.); chenyoulong2@126.com (Y.-L.C.); xchen@wit.edu.cn (X.-B.C.); 2 School of Mathematics and Physics, China University of Geosciences (Wuhan), Wuhan 430074, China; 3 Department of Physics, Wuhan University, Wuhan 430072, China

**Keywords:** plasmon coupling, electromagnetic enhancement, Au nanocrystals, photocatalysis, SERS

## Abstract

Flower-like metallic nanocrystals have shown great potential in the fields of nanophononics and energy conversion owing to their unique optical properties and particular structures. Herein, colloid Au nanoflowers with different numbers of petals were prepared by a steerable template process. The structure-adjustable Au nanoflowers possessed double plasmon resonances, tunable electric fields, and greatly enhanced SERS and photocatalytic activity. In the extinction spectra, Au nanoflowers had a strong electric dipole resonance located around 530 to 550 nm. Meanwhile, a longitudinal plasmon resonance (730~760 nm) was obtained when the number of petals of Au nanoflowers increased to two or more. Numerical simulations verified that the strong electric fields of Au nanoflowers were located at the interface between the Au nanosphere and Au nanopetals, caused by the strong plasmon coupling. They could be further tuned by adding more Au nanopetals. Meanwhile, much stronger electric fields of Au nanoflowers with two or more petals were identified under longitudinal plasmon excitation. With these characteristics, Au nanoflowers showed excellent SERS and photocatalytic performances, which were highly dependent on the number of petals. Four-petal Au nanoflowers possessed the highest SERS activity on detecting Rhodamine B (excited both at 532 and 785 nm) and the strongest photocatalytic activity toward photodegrading methylene blue under visible light irradiation, caused by the strong multi-interfacial plasmon coupling and longitudinal plasmon resonance.

## 1. Introduction

Plasmonic metal nanocrystals have attracted considerable attention and have been widely applied in surface-enhanced Raman spectroscopy (SERS), nonlinear optical applications, photocatalysis, and photothermal conversion [1,2,3,4]. Surface plasmons originate from the collective oscillations of electrons arising on the surface of metals upon electromagnetic excitation. Under plasmon resonance excitation, the electromagnetic fields near the metal nanocrystals are greatly enhanced and the light absorption can be distinctly boosted near the resonant frequency. These advantages match well with the demands of photocatalysis and SERS. Photocatalysis is a green technology that can produce clean solar fuels and remedy environmental problems, and SERS is applied in the super-sensitive detection of biomolecules, pollutants, and pesticides at a very low concentration through their unique vibrational fingerprints [5,6,7,8]. The well-known mechanism behind the plasmonic metal-based SERS and photocatalysis is electromagnetic enhancement, namely hotspots, which refers to the enhancement of the local electromagnetic field as a result of the excitement of surface plasmons by incident light. In the SERS, the electromagnetic field can amplify the vibration by several orders of magnitudes. In photocatalysis, the electromagnetic field can offer abundant hot electrons, increase the speed of charge transfer and separation, and generate a local heating effect, which are very useful to catalytic reactions. Normally, plasmonic metal nanocrystals such as Au, Ag, and Cu have been dominant as the Raman substrates and photocatalysts for highly improved SERS and photocatalysis possessing electromagnetic enhancement, respectively [9,10,11,12]. The local electromagnetic fields of metal nanocrystals highly depend on the morphology, size, composition, and the surrounding dielectric environment [13,14]. The diverse structures and sizes of the same metal nanocrystals usually have entirely different enhancement factors of SERS and photocatalysis caused by the tunable local electromagnetic fields [15,16,17].

The combination of two or more plasmonic metals with an ultrashort distance will produce strong interfacial plasmon coupling, which can generate a much stronger electromagnetic field localization and enhancement than isolated metals can [18,19,20]. The plasmon coupling-based electromagnetic field can offer greater SERS and photocatalytic enhancements than individual metal nanocrystals can. The electromagnetic field generated by interfacial plasmonic coupling can be optimized by changing the size, shape, configuration, and architecture. In addition, for some particular plasmon coupling systems, new plasmonic modes will appear, such as magnetic and longitudinal resonance [21,22,23], which can also promote the SERS and photocatalysis activity. Taking advantage of advanced chemical synthesis and physical fabrication methods, diverse types of metals featuring plasmon coupling have been designed and prepared in the forms of Janus-like structures [24], core–shell motifs [25,26,27], particle-film systems [28,29,30], and self-assembled structures [31,32,33,34]. Meanwhile, some picturesque nanostructures have also been reported, such as nanosnowman [35], nanodumbbells [36], and nanoflowers [37,38,39,40,41]. As a typical plasmon-coupling structure, flower-like structures possess numerous corners, tips, and gaps, offering an ideal platform for plasmon coupling and generating abundant hotspots. Previous works have reported the flower-like Au, Ag, and Au/Ag hybrids acting as Raman substrates and photocatalysts to achieve excellent SERS and photocatalytic performances [38,40,42,43]. In the flower-like structure, the protruding petal is the key factor to achieving strong plasmon coupling or producing new plasmon modes. However, the precise regulation of the petals is usually uncontrollable and has rarely been reported due to the limits of synthetic strategies.

In the present work, colloid Au nanoflowers with different numbers of petals were prepared via a template method, which showed strong plasmon coupling, tunable electric fields, and greatly enhanced SERS and photocatalytic activity. The as-prepared Au nanoflowers had a strong electric dipole resonance located around 530~550 nm in the extinction spectra. When precisely increasing the number of petal of Au nanoflowers to two or more, a new longitudinal plasmon resonance appeared, located around 730~760 nm. The Au nanoflowers possessed tunable numbers of nanopetals, dual plasmon modes, and abundant hotspots, showing better potentials with SERS and photocatalysis than traditional Au nanocrystals, such as Au nanospheres, nanocubes, or nanoplates. On detection of RhB, structure-adjustable Au nanoflowers showed excellent SERS performances, which were highly tuned by varying the number of petals. Four-petal Au nanoflowers possessed the highest SERS activity excited both at 532 and 785 nm. The highest enhancement factor reached 5.3 × 10^8^, and the detection limit reached 10^−12^ M. The numerical simulations verified that the strong electromagnetic field enhancements caused by multi-interfacial plasmon coupling and longitudinal plasmon resonance were responsible for the excellent SERS performances. Additionally, the nanoflowers also showed excellent and petal-number-dependent photocatalytic activity on degrading methylene blue (MB) by NaBH_4_ with light irradiation. Four-petal Au nanoflowers possessed the highest photocatalytic activity, which was 5.86 and 3.78 times those of Au nanospheres and one-petal Au nanoflowers, respectively.

## 2. Methods

### 2.1. Chemicals

Chloroauric acid (99.99%), sodium hydroxide (99.7%), L-ascorbic acid (AA, 99.7%), sodium borohydride (96.0%), lead acetate (99.5%), thioacetamide (TAA, 99.0%), hexamethylenetetramine (HMT, 99.0%), hexadecyltrimethylammonium bromide (CTAB, 99.0%), and hydrochloric acid (36–38%) were purchased from Sinopharm Chemical Reagent Co. Ltd. (Shanghai, China). Deionized water with a resistivity of about 18.25 MΩ·cm was used as the solvent in all experiments.

### 2.2. Synthesis of Au/PbS Hybrids

PbS nanoshells were partially coated on Au nanospheres. The initial CTAB-stabilized Au nanospheres were prepared by a modified seed-mediated method. To synthesize Au/PbS hybrids, 1 mL of AA (0.1 M), 2 mL of CTAB (0.2 M), and 1 mL of HMT (0.1 M) were added to 5 mL of as-prepared Au nanospheres. Then, 0.03 mL of TAA (0.1 M) and 0.01~0.05 mL of lead acetate (0.1 M) were added. The mixture solution was maintained at 85 °C in a vacuum oven for 8 h. The products were centrifuged at 8000 rpm for 5 min and re-dispersed in water for further use.

### 2.3. Synthesis of Au Nanoflowers

Typically, 0.5 mL of HAuCl_4_ (0.01 M), 0.5 mL of AA (0.1 M), and 10 mL of CTAB (0.05 M) were added to 2 mL of as-prepared Au/PbS hybrids. Stirring of the mixture solution was continued at 1000 rpm for 2 h at room temperature. The final products were centrifuged at 8000 rpm for 5 min and re-dispersed in water for further use. To dissolve PbS nanoshells, 0.1 mL of HCl (1 M) was added to 2 mL of as-prepared Au/PbS/Au hybrids. The mixture solution was maintained at 60 °C in a vacuum oven for 2 h. The products were centrifuged at 8000 rpm for 5 min and re-dispersed in water for further use. The number of petals of the Au nanoflowers were adjusted by varying the amount of lead acetate and sulfur source in the process of PbS growth.

### 2.4. Photocatalytic Measurements

Typically, 1 mL of distilled water was mixed with 0.75 mL of 0.4 mM of methylene blue (MB) solution in a quartz cuvette, and 0.25 mL of H_2_O_2_ solution was quickly added. Then, catalysts (including Au nanospheres and Au nanoflowers) were injected rapidly. The reduction of MB was verified by monitoring the decrease in the extinction intensity at 665 nm. For the light-assisted catalytic reduction, a 300 W Xenon lamp was chosen as the light source. An ultraviolet cut-off filter (*λ* > 420 nm) was used to obtain the visible light.

### 2.5. Numerical Simulation

Commercial software (COMSOL Multiphysics) was used for the finite-element method (FEM) simulations. The refractive index of water is 1.33, and refractive indices of Au were taken from Ref. [44]. Perfectly matched layers were used in the simulations. An Au nanosphere was taken to be a sphere with a radius of 10 nm. For the one-petal Au nanoflower, the central Au nanosphere was taken to be a sphere with a radius of 10 nm, and the petal was taken to be an ellipsoid, whose a, b, and c were set as 10, 12, and 14 nm, respectively. For two-, three-, and four-petal Au nanoflowers, the central Au and petals were taken to be spheres with a radius of 10 nm. The excitation light was along the x axis and polarized along the y axis in the calculations.

### 2.6. Sample Characterization

Scanning electron microscopy (SEM) observations were performed with a FEG SEM Sirion 200 (FEI Electronic Optics Company, Hillsboro, OR, USA) operating at an accelerating voltage of 25.0 kV. The SERS spectra were acquired with a laser source with wavelengths of 532 nm (1 mW) and 785 nm (1.5 mW) for 10 s of illumination. Each spectrum represented the average spectrum from three replicates. The extinction spectra were tested by UV-VIS-NIR spectrophotometry (TU1810, Beijing Pgeneral, Beijing, China).

## 3. Results and Discussion

**Figure 1:**
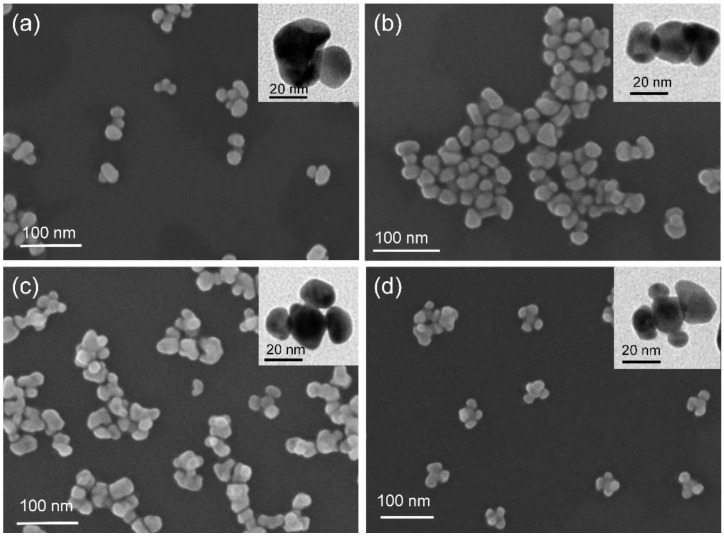
SEM and TEM images of Au nanoflowers with one (**a**), two (**b**), three (**c**), and four (**d**) petals.

The Au nanoflowers with tunable numbers of petals were synthesized via a modified three-step method based on Au nanospheres, which were similarly reported in our previous works [8,12]. In brief, monodispersed Au nanospheres were first prepared by a seed-mediated method. Then, PbS nanoshells were partially coated on Au nanospheres using thioacetamide and lead acetate as a sulfur source and precursor at a high-concentration surfactant, respectively. Subsequently, Au nanoshells were deposited on Au/PbS hybrids to form Au/PbS/Au hybrids. The Au overgrowth started preferentially at the exposed surface of Au nanospheres. Finally, Au nanoflowers were obtained by dissolving the PbS nanoshell off in the presence of hydrochloric acid. The number of petals of the Au nanoflowers was adjusted by varying the amount of lead acetate and sulfur source in the process of PbS growth. Figure 1 displays a set of representative SEM and TEM images of the structure-adjustable Au nanoflowers. The inset shows the corresponding modes. The four types of Au nanocrystals had the same central Au nanospheres, which can be regarded as the flower heart. The overgrown Au shells attached on the Au nanospheres can be seen as flower petals. The central Au nanospheres had uniform size, with average diameters of 20 nm, while the Au flower petals had different sizes ranging from 14 to 45 nm. Noticeably, the number of petals was precisely tuned in the range from one to four (see Figure 1a–d). The Au nanoflowers with tunable numbers of petals possessed uneven surfaces and particle–particle nanogaps, offering a good platform for plasmon coupling.

**Figure 2:**
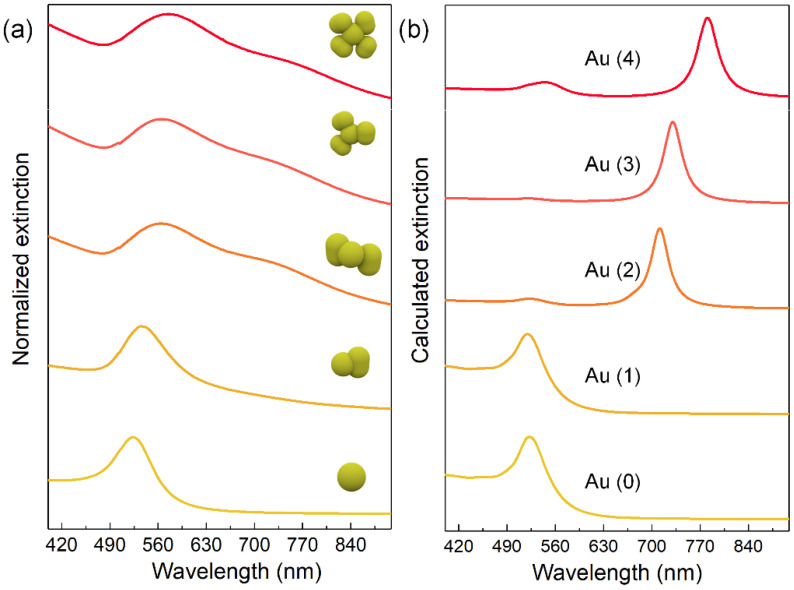
Experimental (**a**) and calculated (**b**) extinction spectra of Au nanospheres and structure-adjustable Au nanoflowers. The Au (0), Au (1), Au (2), Au (3), and Au (4) represent the Au nanosphere and, one-, two-, three-, and four-petal Au nanoflowers, respectively.

The plasmon resonances of the Au nanoflowers with different numbers of petals were investigated both experimentally and theoretically. Figure 2a shows the experimental extinction spectra of Au nanospheres and Au nanoflowers with different numbers of petals. The starting Au nanospheres displayed sharp plasmon peaks around 530 nm, originating from the electric dipole resonance. For one-petal Au nanoflowers, the dipole resonance peak redshifted and broadened, probably caused by the increased size and the plasmon coupling between the Au core and Au petal. Noticeably, the as-prepared Au nanoflowers with two or more petals exhibited two resonance peaks in the extinction spectra. The major peak at the high-energy side could be assigned to the electric dipole mode coming from the intrinsic resonance of Au. Similarly, this mode redshifted and broadened with the number of petals. The weak shoulder (around 730~760 nm) could be attributed to the multipolar coupling between the Au nanospheres and Au petals induced by the longitudinal polarization excitation. This plasmon resonance also redshifted with the number of petals. Figure 2b displays the calculated extinction spectra of the Au nanospheres and Au nanoflowers with different numbers of petals. The calculation results matched well with the key peak positions observed in the experiments. Electric dipole resonances ranging from 528 to 545 nm for Au nanospheres and Au nanoflowers were obtained. Meanwhile, the longitudinal plasmon resonances of Au nanoflowers with two or more petals were also observed. The calculated variation trends also matched well with the experimental results, with both plasmon modes redshifting with the number of petals.

**Figure 3:**
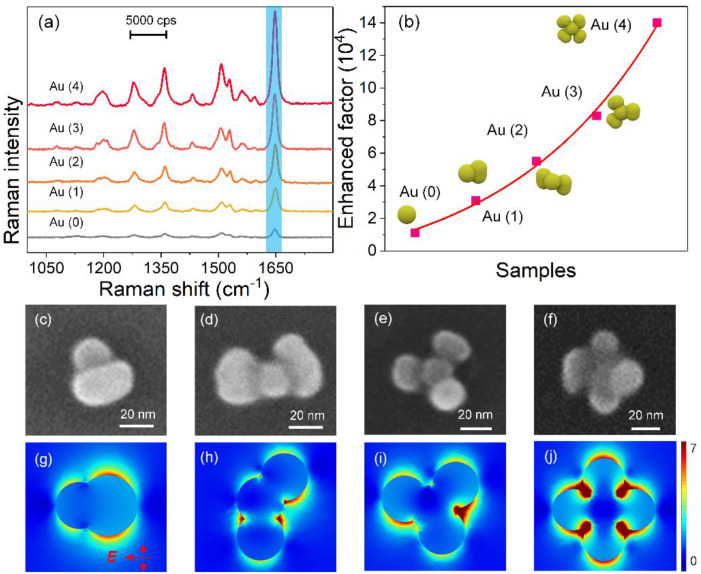
(**a**) Raman spectra of RhB (10^−6^ M) absorbed on Au nanospheres and structure-adjustable Au nanoflowers under a 532 nm laser excitation. (**b**) Calculated SERS EFs of RhB (10^−6^ M) at 1647 cm^−1^ in the presences of Au nanospheres and nanoflowers. SEM images and calculated electric field distributions (excited at 532 nm) of one—(**c**,**g**), two—(**d**,**h**), three—(**e**,**i**), and four—(**f**,**j**) petal Au nanoflowers.

To apply the tunable plasmon coupling for SERS application, we used these Au nanoflowers as substrates to detect RhB. The SERS sensitivities of the Au nanoflowers were first investigated using the excitation of a 532 nm laser, which matched well with the electric dipole resonance. Figure 3a displays the Raman spectra of RhB absorbed on the Au nanospheres and Au nanoflowers with different numbers of petals. The Au nanoflowers showed much higher Raman intensities than Au nanospheres did, and the SERS responses were greatly enhanced with the numbers of petals. Noticeably, four-petal Au nanoflowers possessed the highest Raman signal. In order to quantitatively compare SERS activities of the above samples, enhancement factors (EF) were calculated with the intensity of 1647 cm^−1^ (10^−6^ M) based on the magnification of Raman intensity compared with that on a glass slide (Figure 3b). The EFs of Au nanoflowers were much higher than those of Au nanospheres, and they almost increased exponentially with the number of petals. Four-petal Au nanoflowers had the largest EF, reaching 1.4 × 10^5^, which was 14 and 4.5 times those of Au nanospheres and one-petal Au nanoflowers, respectively. To reveal the physical mechanism behind the improved SERS, the local electric fields of the Au nanoflowers were calculated. Figure 3c–j show the SEM images and electric field distribution of Au nanoflowers with different numbers of petals. The electric field distributions were calculated at the excitation of 532 nm. The Au nanoflowers showed structure-dependent electric field distributions. In particular, the one-petal Au nanoflower displayed more hotspots than those of the Au nanosphere (Supplementary Materials, Figure S1), indicating that the electromagnetic field was the main enhanced factor for the enhanced SERS. For Au nanoflowers with two petals, strong electric fields located around the interface between the Au core and Au petals were observed, coming from the strong plasmon coupling. The numbers of hotspots increased with the number of petals. As shown in Figure 3j, the four-petal Au nanoflower showed the strongest interfacial electric fields and the most abundant hotspots, caused by the multi-interfacial plasmon coupling between the Au core and petals. The Au nanoflowers with four petals displayed the highest SERS signal, most abundant hotspots, and strongest electric field, demonstrating that the multi-interfacial plasmon coupling played a key role in the improved SERS performance.

**Figure 4:**
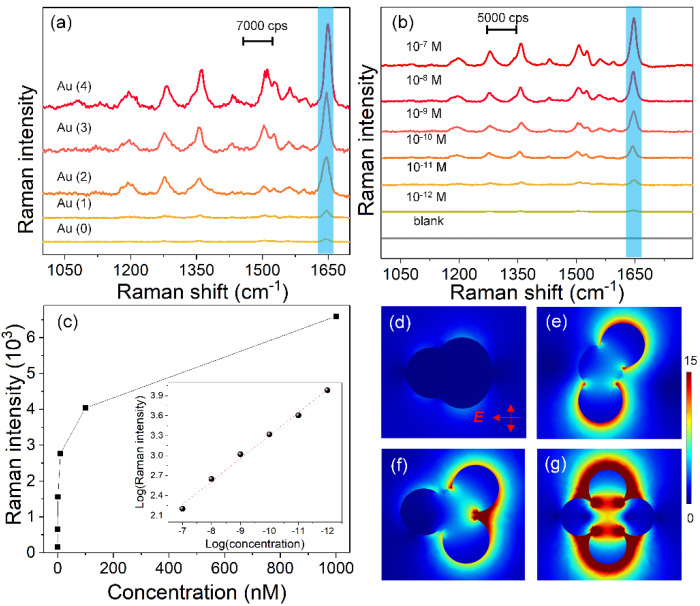
(**a**) Raman spectra of RhB (10^−6^ M) absorbed on Au nanospheres and structure-adjustable Au nanoflowers under a 785 nm laser excitation. (**b**) Concentration-dependent SERS spectra of RhB at various concentrations absorbed on four-petal Au nanoflowers. (**c**) Plots of the Raman intensity at 1647 cm^−1^ as a function of RhB concentration, where the inset figure represents the linear relationship from 10^−7^ to 10^−12^ M. (**d**–**g**) Calculated electric field distributions of Au nanoflowers excited at 785 nm.

In order to study the enhancement of longitudinal plasmon resonance to SERS, Raman measurements of the above-mentioned samples were performed under an excitation of 785 nm. Figure 4a displays the Raman spectra of RhB (10^−6^ M) absorbed on Au nanospheres and Au nanoflowers. Au nanospheres and one-petal Au nanoflowers showed very weak SERS signals. This is because they have very weak absorption around 785 nm and cannot produce enough electromagnetic enhancement to amplify the Raman signals. The SERS responses of Au nanoflowers were still highly related to the number of petals. Au nanoflowers with four petals exhibited the highest SERS activity compared with Au nanospheres and the other three kinds of Au nanoflowers. The EF (calculated at 1647 cm^−1^, 10^−6^ M) of four-petal Au nanoflowers reached 1.9 × 10^5^, which was about 42 and 14 times those of the Au nanospheres and one-petal Au nanoflowers, respectively (see Figure S2). In addition, the EF at 785 nm of four-petal Au nanoflowers was much higher than that excited at 532 nm, with the enhancement reaching 1.4 fold. Concentration-dependent SERS measurements of RhB (from 10^−7^ to 10^−12^ M) absorbed on four-petal Au nanoflowers were performed to identify the SERS detection limit. As shown in Figure 4b, even when the concentration of RhB solution decreased to 10^−12^ M, the SERS signals were still identifiable. The maximum EF was calculated as 5.3 × 10^8^ (10^−12^ M, at 1647 cm^−1^). The variation curve based on the concentration of RhB and Raman intensity at 1647 cm^−1^ is shown in Figure 4c. The logarithmic curve showed good linearity as the concentrations changed from 10^−7^ to 10^−12^ M (inset), indicating the high sensitivity and wide quantitation range of four-petal Au nanoflowers. The electric field distributions excited at 785 nm of these Au nanoflowers were calculated to explain the physical mechanism of the enhanced SERS performance. As shown in Figure 4d,e, the one-petal Au nanoflower had a very weak electric field, while Au nanoflowers with two or more petals showed a much stronger electric field along the polarized directions, caused by the longitudinal plasmon resonance excitation. Most interestingly, the electric fields were much stronger than those excited at 532 nm, matching well with the Raman results, demonstrating that longitudinal plasmon resonances had a higher enhancement on SERS. The four-petal Au nanoflowers offered two possible vertical directions of longitudinal excitation and multi-interfacial plasmon coupling, thereby producing the strongest electromagnetic enhancement, finally greatly amplifying the SERS signal.

**Figure 5:**
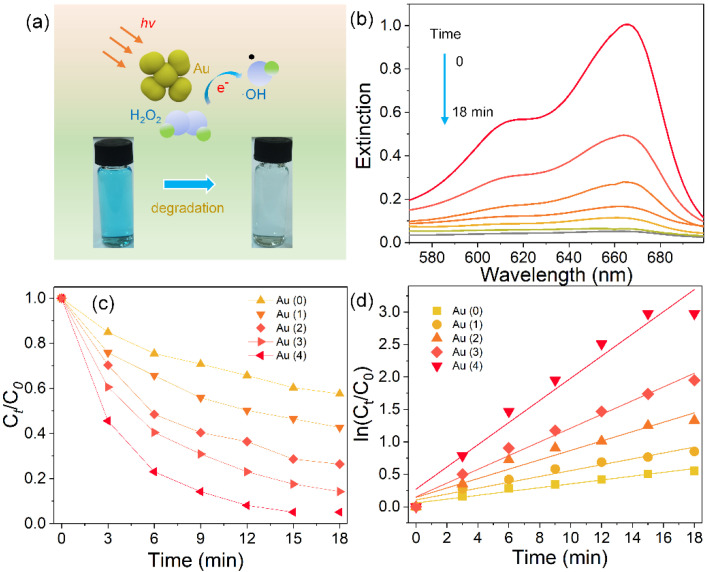
(**a**) Schematic illustration of the degradation of MB under light irradiation in the presence of Au nanoflowers and H_2_O_2_. (**b**) Extinction spectra of MB recorded at different reaction time points in the presence of four-petal Au nanoflowers. Photocatalytic degradation curves (**c**) and logarithm of the absorbance at 665 nm versus reduction time (**d**) of MB over different catalysts.

The as-prepared Au nanoflowers had a rough surface, strong light absorption ranging from the visible to infrared region, and tunable electric field enhancement, showing great potential in photocatalysis. We adopted the reduction of MB by H_2_O_2_ with light irradiation (*λ* > 420 nm) as a model reaction to evaluate the catalytic activity of Au nanoflowers. Figure 5a schematically displays the MB degradation via Fenton-like reaction in the presence of Au and H_2_O_2_. Briefly, the Au can activate H_2_O_2_, thus generating hydroxyl radicals via a reversible cycle (Au^0^→Au^+^→Au^0^), and then reducing MB. These reactions can be significantly enhanced when irradiated with light due to the plasmon-induced light absorption and hot electrons. The concentration of MB was monitored by recording the extinction intensity at 665 nm. Figure 5b shows a typical set of extinction spectra recorded at different time points of the reaction when four-petal Au nanoflowers were used as a catalyst. The MB was rapidly reduced in 18 min as observed in the time-dependent UV-visible absorption spectra. We compared the photocatalytic activity of Au and structure-adjustable Au nanospheres. Figure 5b shows that the Au nanoflowers had a much higher photocatalytic activity than Au nanospheres did, and the photocatalytic rate could be further optimized by increasing the number of petals of Au nanoflowers. The apparent reaction rate constants were calculated by plotting In(*C_t_/C*_0_) as a function of time, which are shown in Figure 5d. The *C*_0_ and *C_t_* refer to initial and real-time extinction intensities at 665 nm of MB, respectively. Four-petal Au nanoflowers exhibited the fastest photoactivity with rate constant *k* = 0.17 min^−1^, which was 5.86 and 3.78 times those of Au nanospheres and one-petal Au nanoflowers, respectively. This is because the four-petal Au nanoflowers exhibited strong two-mode plasmon resonance, which can act as a light-harvesting unit, thus leading to intense light absorption in the visible and near-infrared regions. In addition, the strong electric field caused by the multi-interfacial plasmon coupling can promote the generation and transfer of hot electrons, eventually quickening the photocatalytic reaction.

## 4. Conclusions

In summary, we synthesized colloidal Au nanoflowers with a steerable number of petals and investigated their strong plasmon coupling, tunable electric field, and largely enhanced SERS activity. The Au nanoflowers had a strong electric dipole resonance located from 530 to 550 nm, and a longitudinal plasmon resonance (730~760 nm) was observed when the number of petals of Au nanoflowers increased to two or more. Numerical simulations verified that the strong electric fields of Au nanoflowers were located at the interface between the Au nanosphere and Au nanopetals, caused by the strong plasmon coupling. Meanwhile, the stronger electric field of Au nanoflowers with two or more petals were identified under longitudinal plasmon excitation. The Au nanoflowers showed highly improved SERS and photocatalytic activity. Four-petal Au nanoflowers displayed the highest SERS signals on detecting RhB activity excited at both 532 and 785 nm. Meanwhile, four-petal Au nanoflowers exhibited the fastest photoactivity, which was 5.86 and 3.78 times those of Au nanospheres and one-petal Au nanoflowers, respectively, toward the Fenton-like reduction of MB. These findings provide inspiration for the design of plasmonic antennas, and the materials will find diverse promising applications in the fields of photodetection and biological imaging.

## Data Availability

No new data were created or analyzed in this study. Data sharing is not applicable to this article.

## Supplementary Materials

The following are available online at https://www.mdpi.com/article/10.3390/nano11092176/s1, Figure S1: Morphology and optical properties of Au nanospheres, Figure S2: Calculated SERS of Au nanospheres and flowers excited at 785 nm.
