# Supplementary material for: Dual Plasmon Resonances and Tunable Electric Field in Structure-Adjustable Au Nanoflowers for Improved SERS and Photocatalysis
Source: Nanomaterials (Basel). 2021 Aug 25;11(9):2176. doi: 10.3390/nano11092176 (PMC8466837; doi:10.3390/nano11092176)
Supplement: Supplementary file 1 [file nanomaterials-11-02176-s001.zip › nanomaterials-1355031-supplementary.pdf]

Supporting Materials for

Dual Plasmon Resonances and Tunable Electric Field in Structure-Adjustable Au Nanoflowers for Improved SERS and Photocatalysis

Yi-Xin Zhao [1], Hao-Sen Kang [1], Wen-Qin Zhao [1], You-Long Chen [1], Liang Ma [1,*], Si-Jing Ding [2,*], Xiang-Bai Chen [1] and Qu-Quan Wang [3,*]

[1] Hubei Key Laboratory of Optical Information and Pattern Recognition, Wuhan Institute of Technology, Wuhan 430205, China; yujin65536@126.com (Y.-X.Z.); kanghaosen2021@126.com (H.-S.K.); zwq1913256258@126.com (W.-Q.Z.); chenyoulong2@126.com (Y.-L.C.); xchen@wit.edu.cn (X.-B.C.)

[2] School of Mathematics and Physics, China University of Geosciences (Wuhan), Wuhan 430074, China

[3] Department of Physics, Wuhan University, Wuhan 430072, China

* Correspondence: maliang@wit.edu.cn (L.M.); dingsijing@cug.edu.cn (S.-J.D.); qqwang@whu.edu.cn (Q.-Q.W.)

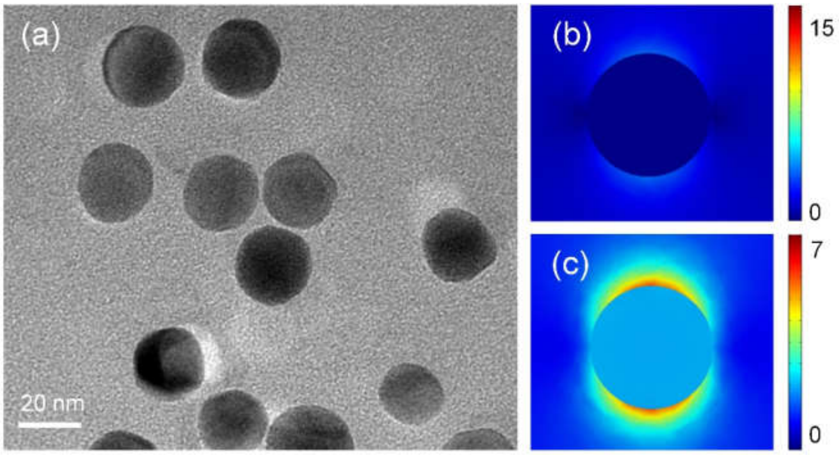

Figure S1. (**a**) TEM image of Au nanospheres. Local electric field distributions of Au nanospheres, excited at 785 (**b**) and 532 nm (**c**).

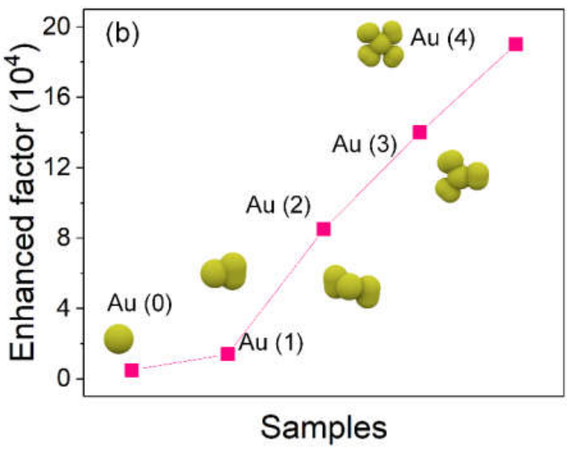

Figure S2. Calculated SERS EFs of RhB (10^{-6} M) at 1647 cm^{-1} in the presences of Au nanospheres and nanoflowers excited at 785 nm.
